# Environmental Sampling as a Low-Technology Method for Surveillance of Foot-and-Mouth Disease Virus in an Area of Endemicity

**DOI:** 10.1128/AEM.00686-18

**Published:** 2018-08-01

**Authors:** Claire Colenutt, Emma Brown, Noel Nelson, Jemma Wadsworth, Jenny Maud, Bishnu Adhikari, Sharmila Chapagain Kafle, Mukul Upadhyaya, Samjhana Kafle Pandey, David J. Paton, Keith Sumption, Simon Gubbins

**Affiliations:** a The Pirbright Institute, Pirbright, Surrey, United Kingdom; b The Met Office, Exeter, Devon, United Kingdom; c European Commission for the Control of Foot-and-Mouth Disease (EuFMD), Food and Agriculture Organization of the United Nations (FAO), Rome, Italy; d Food and Agriculture Organization of the United Nations, Nepal Country Office, Kathmandu, Nepal; e FMD and TADs Laboratory, Department of Livestock Services, Ministry of Livestock Development, Kathmandu, Nepal; f Veterinary Epidemiology Centre, Department of Livestock Services, Ministry of Livestock Development, Kathmandu, Nepal; g Directorate of Animal Health, Department of Livestock Services, Ministry of Livestock Development, Kathmandu, Nepal; Rutgers, The State University of New Jersey

**Keywords:** environmental surveillance, foot-and-mouth disease, FMD, viral detection, rRT-PCR, foot-and-mouth disease virus

## Abstract

Environmental sampling enables disease surveillance beyond regular investigation of observed clinical cases, extending data on the circulation of a pathogen in a specific area. Developing straightforward, low-technology methods suitable for use under field conditions is key to the inclusion of such approaches alongside traditional surveillance techniques. Foot-and-mouth disease virus (FMDV) is an economically important livestock pathogen, affecting cloven-hoofed livestock in many countries. Countries with FMDV face severe trade restrictions, and infections can have long-term effects on the productivity of affected animals. Environmental contamination by the virus in excretions and secretions from infected individuals promotes transmission but also presents an opportunity for noninvasive sample collection, facilitating diagnostic and surveillance activities. We present environmental sampling methods that have been tested in the Kathmandu Valley, Nepal, where FMDV is endemic. A total of nine sites were visited and sampled between November 2016 and November 2017. Environmental swabs collected from sites with reported outbreaks of FMD were used to demonstrate successful detection of FMDV RNA from the environment. The development of methods that can reliably detect FMDV RNA in the environment is significant, since this possibility extends the toolbox available for surveillance for this disease. Similar methods have already been deployed in the effort to eradicate polio, and with FMDV, such methods could easily be deployed in the event of an outbreak to provide additional resources for detection that would relieve pressure on veterinary services. The development of low-technology, straightforward surveillance methods such as these can support a robust response to outbreaks.

**IMPORTANCE** Prompt confirmation and diagnosis of disease are key factors in controlling outbreaks. The development of sampling techniques to detect FMDV RNA from the environment will extend the tool kit available for the surveillance of this pathogen. The methods presented in this article broaden surveillance opportunities using accessible techniques. Pairing these methods with existing and novel diagnostic tests will improve the capability for rapid detection of outbreaks and implementation of timely interventions to control outbreaks. In areas of endemicity, these methods can be implemented to extend surveillance beyond the investigation of clinical cases, providing additional data for the assessment of virus circulation in specific areas.

## Abstract (precis)

Prompt confirmation and diagnosis of disease are key factors in controlling outbreaks. The development of sampling techniques to detect FMDV RNA from the environment will extend the tool kit available for the surveillance of this pathogen. The methods presented in this article broaden surveillance opportunities using accessible techniques. Pairing these methods with existing and novel diagnostic tests will improve the capability for rapid detection of outbreaks and implementation of timely interventions to control outbreaks. In areas of endemicity, these methods can be implemented to extend surveillance beyond the investigation of clinical cases, providing additional data for the assessment of virus circulation in specific areas.

## INTRODUCTION

Foot-and-mouth disease (FMD) is a globally important disease of cloven-hoofed livestock and wildlife species. Transmission occurs primarily through direct contact between infected and susceptible individuals, but indirect transmission of the virus in animal products, on fomites, or in aerosols can facilitate longer-distance transmission events (1). The disease can be controlled through vaccination, culling of infected animals or herds, and implementation of strict decontamination and quarantine regulations when outbreaks occur. FMD remains endemic in parts of Africa, Asia, and the Middle East (2). Europe, North and Central America, and the Pacific nations are free of FMD. The presence of FMD can cause significant economic costs (3) through direct production losses in regions of endemicity, restrictions on international trade, and the associated costs of control measures.

FMD is notifiable to the World Organization for Animal Health (OIE). Detection of FMD virus (FMDV)-infected farms relies largely on observation and the reporting of clinical signs. Clinical samples are then sent to specialized laboratories to confirm the presence of FMDV by laboratory tests (4). Surveillance to determine the extent of virus circulation is carried out primarily using seroprevalence surveys (5).

Environmental sampling is commonly used for the detection of circulating pathogens outside the scope of clinical observations (6, 7). FMDV can be found in all secretions and excretions from acutely infected animals, including exhaled air, saliva, urine, and feces, resulting in contamination of the environment around infected individuals. FMDV can remain in the environment for long periods (8), although the viability of virions in the environment depends on specific conditions, since the virus is sensitive to drying out, pH extremes, and high temperatures (9, 10). With appropriate sampling and testing techniques, detection of FMDV from contaminated environments is a plausible option for surveillance.

We report the development and testing of sampling methods for the detection of FMDV from the environment. These methods are presented as additional low-technology and low-cost surveillance tools for FMDV. A preliminary evaluation of these sampling methods took place in the Kathmandu Valley, Nepal, as an adjunct to training courses in FMD outbreak investigation that were provided by the European Commission for the Control of Foot-and-Mouth Disease (EuFMD). FMDV is endemic in susceptible animal populations in Nepal, where subsistence farming with small-scale livestock production is common practice.

## RESULTS

### Study area.

**FIG 1:**
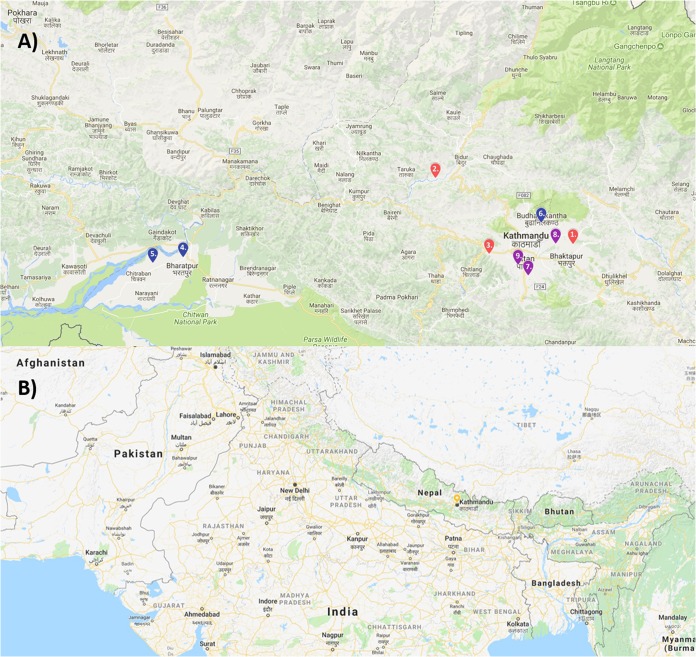
Map showing locations of sampling sites. (A) Locations of sampling sites used for collection of samples. Sites are color coded for the time of the visit: November 2016 (red), April 2017 (blue), or November 2017 (magenta). (B) Kathmandu (yellow pin) in relation to surrounding countries. Map data ©2018 Google.

**FIG 2:**
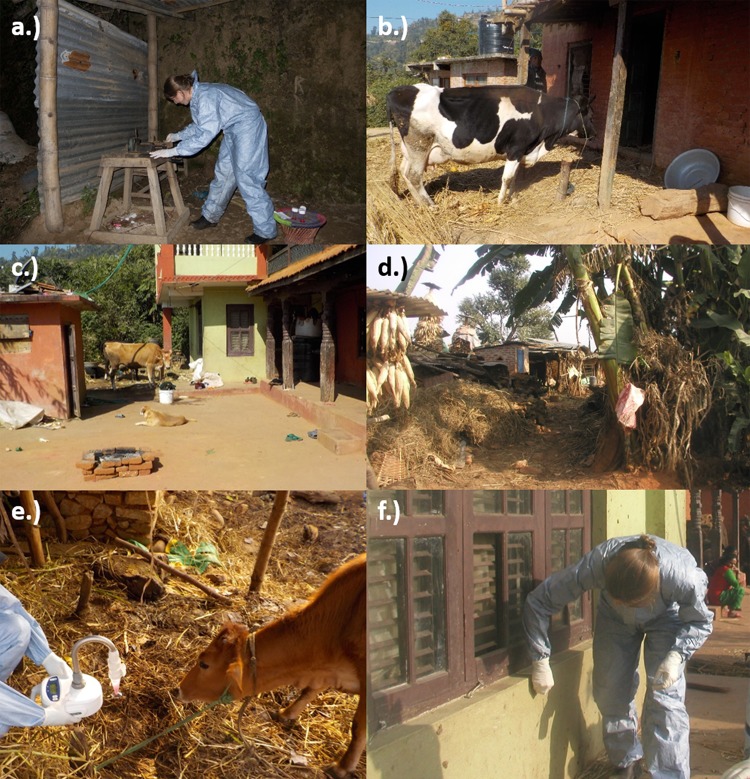
Sampling sites and collection. (a) Environmental sampling at the milk collection point at site 1. (b) Courtyard at household 6, site 1. (c) Courtyard at household 5, site 2. (d) Main pathway through the village at site 2. (e) Air sampling at site 2. (f) Environmental sampling at household 5, site 1.

Outbreaks at nine different sites were investigated and used for sample collection over three visits. The first three sites (sites 1, 2, and 3) were visited in late November to early December 2016; sites 4, 5, and 6 were visited in April and May 2017; and sites 7, 8, and 9 were visited in November 2017 (Fig. 1). Sites generally comprised houses arranged in villages, with paths linking premises and shared grazing or forage collection sites and farmed land around the villages (Fig. 2). In terms of FMDV-susceptible species, most households kept at least one cow or water buffalo (Bubalus bubalis) and several goats (Table 1), with cattle and buffalo frequently tethered. Site 3 was much larger than the others, housing 45 cattle and a larger community of people who worked in and around the farm.

**TABLE 1:** Summary of households visited and samples taken during the study

Site	Household	No. of FMD cases	Lesion age (days)^*a*^	No. of positive samples/total no.			Comments
				Environmental	Aerosol	Oral	
1	1	1 cow	6	1/4	2/2	1/1	4 goats also present
1	1 (revisited after 5 days)	1 cow	11	2/27	NS^*b*^	0/1	
1	2	1 cow	5–6	2/4	0/1	NS	Calf and goats also present
2	3	2 cows	4–5; >10	6/12	1/3	1/1	Calf also present
2	4	1 cow	1–2	NS	1/1	1/1	
1	5	2 cows	1–2; 1	8/11	3/3	2/2	
1	5 (revisited after 2 days)	2 cows	3–4; 3	10/18	NS	2/2	Environmental swabs from surfaces similar to those on first visit
1	6	2 cows	3–4; 4–5	8/12	NS	2/2	Calf also present
1	Milk collection point	None		1/4	NS	NS	No livestock present
2	7	None	No lesions	2/12	NS	NS	3 buffalo, unaffected by FMD; next door to household 8
2	8	2 buffalo	4–5	5/11	NS	1/2	Calf also affected; goats also present
2	9	3 buffalo, 1 cow	All >10	3/12	NS	NS	
2	10	1 buffalo	No lesions	1/11	NS	0/1	Possible preclinical case: 1 buffalo (out of 4) had a raised temp
3	11	40	>20	15/32	NS	NS	40/45 cattle had shown clinical signs 20 days previously
4	12	5 cows	>21	4/4	NS	NS	
4	13	2 cows	>21	0/4	NS	NS	
5	14	4 cows	4	3/4	NS	1/1	1 unaffected buffalo also present
5	14 (revisited after 3 days)	4 cows	7	4/4	NS	NS	
5	15	1 cow	4–5	1/2	NS	1/1	
5	16	5 cows	No lesions	4/4	NS	NS	Owner reported that animals were just developing illness
6	17	1 cow	3–4	4/4	NS	NS	
6	18	3 cows	2; 4; 4	6/8	NS	NS	10 cattle in total kept in household
6	19	2 cows	3–4	4/4	NS	NS	17 cattle in total kept in household
7	20	1 cow	20	5/15	0/1	NS	Households 20 and 21 share a shed where animals are housed overnight
7	21	3 cows	>20	3/14	0/1	NS	
7	21 (revisited after 7 days)	3 cows	>27	1/8	NS	NS	
7	22	2 cows	12; 14	16/23	0/1	0/1	
8	23	19 cows	>28	0/4	NS	NS	Mild cases of FMD reported, 19 cows in total
9	24	3 cows	4; no lesions	8/8	NS	NS	Total of 6 cows present; a single cow had mouth and foot lesions, 2 cows displayed a drop in milk production but no other clinical signs

a Only cattle or buffalo were examined clinically for signs of foot-and-mouth disease.

b NS, no sample taken.

### Outbreak description.

Sites 1 and 2 had active outbreaks. The households visited for sampling at these sites comprised cases ranging from 1 to 6 and 2 to 10 days old, respectively. The outbreak at the farm at site 3 was older and no longer producing new cases. The livestock owner at site 3 reported infection in 40 out of 45 cattle approximately 20 days prior to the sampling visit. The cases reported at site 4 were approximately 3 weeks old, and no new cases were being generated in the outbreak. Site 5 had an active outbreak, with cases from 4 to 7 days old at inspected households. Two households at site 6 had 10 or more cattle, although not all animals displayed clinical signs of FMD. The cases at site 6 at the time of the visit ranged from 2 to 4 days old. Site 7 had older cases of FMD, from an outbreak that began approximately 3 weeks before the site was visited for sample collection. Site 8, a dairy farm with 19 cows, reported mild cases of FMD approximately 1 month before samples were collected. Samples were collected from a single household at site 9, where a total of six cows were present; one cow had 4-day-old lesions in the feet and mouth, and a further two cows were reported to have a drop in milk production.

### Environmental sampling.

Environmental swab samples were collected from all sites. At site 1, four households were sampled, and the percentage of positive environmental swab samples taken from a household ranged from 25% to 73% on the initial visit (Table 1). Two households at the site were revisited several days later. In each case, more samples were collected on the second visit, but the overall percentage of FMDV RNA-positive samples decreased (Table 1). Environmental sampling was also carried out at a milk collection point that serves the households at site 1. The collection point was located on the main track through the village and consisted of an open wooden structure housing a table with weighing scales. No livestock were kept at this location. A total of four swabs were collected, one of which was positive for FMDV RNA.

At site 2, samples were collected from six households. The proportion of FMDV RNA-positive environmental swabs collected from households where clinical cases of FMDV had been confirmed ranged from 25% to 45% (Table 1). Samples were also collected from two households that did not have animals displaying clinical signs of FMD. One (household 7) was next door to a household with infections but had no cattle displaying clinical signs that would lead to suspicion of FMDV infection. The other household (household 10) had a suspected preclinical case, with one buffalo (out of four) displaying a raised temperature, but with no obvious lesions on either the feet or the mouth. Environmental swab samples were collected from both properties; of these, 17% and 9% were positive for FMDV RNA, respectively (Table 1, households 7 and 10).

At site 3, 47% of environmental samples collected were positive for FMDV RNA (Table 1).

Two households were visited at site 4. These had cases that were several weeks old (Table 1). All the samples collected at household 12, which had 5 cows present, were positive for FMDV RNA, but no viral RNA was recovered from samples collected at household 13.

At site 5, three households were visited to collect samples, and the proportion of FMDV RNA-positive environmental samples ranged from 50% to 100% (Table 1). At household 16, the owner reported that the cattle were just developing illness, although the case history is uncertain, since lesions approximately 5 days old had been reported 1 month earlier.

Site 6 had two households where 10 or more cattle were kept on the premises. Not all of the animals at each household were affected by FMDV on clinical inspection (Table 1), but FMDV RNA was detected in environmental samples from both premises (75% and 100% at households 18 and 19, respectively).

The outbreak at site 7 was no longer producing new cases, and the households that were visited had cases that ranged from 12 to >20 days old. FMDV RNA was recovered from samples from all three households, although the proportion of positive samples decreased when household 21 was revisited after 1 week (Table 1).

Sites 8 and 9 each comprised a single household. Site 8 was a dairy farm that had reported mild cases of FMDV approximately 1 month before the sampling visit took place. Nineteen cattle were present, but the number affected by the FMDV outbreak was not reported. No positive samples were recovered from this household (Table 1). At site 9, six cattle were present; lesions were found in one animal, and a drop in milk production was reported for two others. All samples collected from site 9 (household 24) were positive for FMDV RNA.

**FIG 3:**
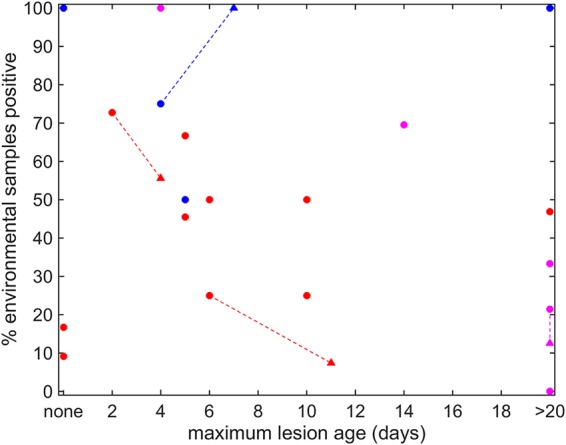
Lesion age versus percentage of positive environmental samples at each farm site. The maximum lesion age was plotted against the percentage of positive samples detected at each household. Lesion ages were based on clinical examination of animals by experienced veterinarians. The different colors indicate the dates of the visits to the household: November 2016 (red), April 2017 (blue), or November 2017 (magenta). Where a household was visited twice, the result for the second visit is indicated by a triangle and is linked to the result for the first visit (indicated by a circle) by a dashed line.

FMDV RNA was detected in environmental samples from premises with animals at all stages of clinical disease, including uninfected, suspected preclinical, clinical, and recovering cattle. The lesion age was compared to the percentage of positive samples collected at each site (Fig. 3). When lesion ages were categorized as fresh (1 to 5 days), healing (6 to 10 days), and old (>10 days), there was a significantly higher proportion of positive samples for households with fresh lesions than for those with old lesions (*P* = 0.02).

**FIG 4:**
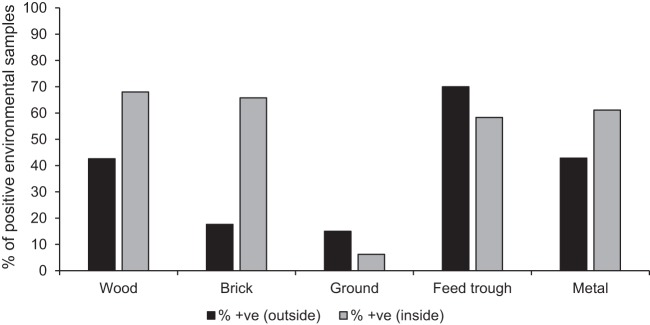
Comparison of samples taken inside with those taken outside. Shown is the percentage of samples positive for FMD viral RNA per sampling surface type. Solid bars show percentages of FMDV RNA-positive samples collected outside, in farm courtyards (from wood [*n* = 54], brick [*n* = 17], the ground [*n* = 20], feed troughs [*n* = 10], and metal [*n* = 14]), and shaded bars show percentages of FMDV RNA-positive samples collected inside cattle barns (from wood [*n* = 5], brick [*n* = 38], the ground [*n* = 16], feed troughs [*n* = 12], and metal [*n* = 18]).

Environmental swabs were collected either in the courtyard of the premises, where animals are kept during the day, or from inside cattle sheds. When the results were assessed by these locations (Fig. 4), samples collected from solid surfaces inside cattle sheds were more likely to be positive for FMDV RNA than samples collected in the household courtyard, except for swabs collected from feed troughs. The differences in the frequency of positive samples between specific sample types and locations were statistically significant for brick inside compared with brick outside (*P* = 0.04) and for brick inside compared with the ground inside (*P* = 0.04).

At sites 1, 2, and 3, a total of 19 fresh fecal samples were collected from the ground, where a specimen could be visually linked to a known clinically affected animal. None of these samples were positive for FMDV RNA, and because of this, further samples of this type were not collected on subsequent visits.

### Oral swabs.

To supplement the diagnosis of FMDV by clinical examination, oral swabs were collected from cattle on most of the farms with recently affected animals, representing half of the households visited. At least one oral swab per household was positive for FMDV RNA. Households 10 and 22 were exceptions; household 10 had a suspected preclinical case, and the lesions in cattle at household 22 were >10 days old (Table 1).

### Aerosol sampling.

In addition to environmental swab samples, ambient air samples were collected in close proximity to infected cattle at five households at sites 1 and 2. Since it was not possible to collect aerosol samples at all sites, these samples are reported separately from the environmental swab samples. Positive aerosol samples were obtained from households 1, 3, 4, and 5 (Table 1). Aerosol samples were also collected at the three households at site 7. No FMDV RNA was recovered from these samples (Table 1).

### Sequencing.

The genomic regions encoding the VP1 capsid protein (the main determinant of serotype) were sequenced for four environmental samples, selected for having cycle threshold (*C_T_*) values below 28. Three of the samples were from household 6 (at site 1), and the fourth sample was from household 4 (at site 2). Sequence data were obtained from a single sample, with the lowest *C_T_* value (22.8). This sample was from household 6, where two cattle were in the early clinical stages of FMD. Three oral samples were collected over two visits to household 6. Sequence data were also obtained for the VP1 regions of these samples, which directly matched the sequence from the environmental sample. A BLAST search of the sequence data confirmed the virus to belong to the O/ME-SA/Ind-2001 lineage, which is reported to circulate in Nepal.

## DISCUSSION

The methods presented in this paper enable the detection of FMD viral RNA from a contaminated environment. Successful isolation of viral RNA was possible from households with animals at different stages of infection, from the preclinical stage of infection to recovery, with animals no longer displaying clinical signs of disease. This demonstrates that even in the absence of clinical signs of disease, the environment is able to serve as a source for detection of viral RNA. Detection of FMD viral RNA from the environment at sites such as household 7, which neighbored an infected household but had no animals suspected of having an FMDV infection, and the milk collection point at site 1 demonstrate that it is not necessary to have animals with clinical disease present in order to detect FMDV genetic material from the environment.

FMDV can remain viable in the environment for long periods, depending on the initial concentration of contamination, environmental conditions, and the material on which the virus is deposited (8–10). The sample collection method used in this study meant that only viral RNA was detected in samples, and infectious viral titers could not be assessed. Developing protocols that enable infectious virus to be reliably detected from field samples is of particular interest, since a contaminated environment can also contribute to the transmission of FMDV (11). The survival of FMDV can be assessed using virus isolation techniques to monitor infectious titers, but there is little information about how long viral RNA can remain detectable in the environment.

Detection of FMDV in environmental swabs was more likely at households with fresh cases of FMDV (1 to 5 days old) than at those with cases categorized as old (>10 days old). This relationship is emphasized by the reduction in the percentage of positive samples detected when households were revisited several days after the initial samples were collected. Only household 14 had an increased detection rate on the second visit, but this is most likely due to the low number of samples collected at each visit (*n* = 4) (Table 1). Despite the reduced chance of detection from older cases, confirmation of the presence of FMDV RNA in the environment at households with cases >10 days old is a useful tool for surveillance. Affected animals may no longer be present at the household, or clinical signs may be difficult to distinguish from those of other diseases. Utilizing environmental sampling could enable surveillance to take place after outbreaks have been resolved, in the absence of reliable reporting of cases. Determining how long after an outbreak FMDV RNA will still be detectable in the environment will allow guidelines to be produced on when environmental sampling can be utilized efficiently. To establish such protocols, the work presented in this study needs to be expanded upon, applying these methods at additional FMDV outbreak sites. The use of environmental sampling methods could also assist in advising on restocking timings once FMDV outbreaks have been resolved, if depopulation is used as a control measure. The association seen between the likelihood of detection from environmental samples and lesion age reflects the basic relationship between the stage of disease and the level of FMDV replication. However, detection from the environment will also be affected by factors such as the initial concentration of environmental contamination, the number of infected animals on the premises, the length of time infected livestock were shedding virus, and meteorological conditions such as temperature and humidity levels. These factors will influence the duration of environmental contamination and the likelihood of detection of the virus in the environment. Reports on environmental survival of FMDV are limited (8), but further application of environmental sampling methods will provide more-detailed information.

Analysis of specific sample types and of the locations on the premises from which positive samples were collected demonstrates that in general, sampling inside a cattle shed will be more likely to generate samples positive for FMDV RNA on an infected premises than sampling outside. This finding may be related to factors that affect virus survival in the environment. Inside a cattle shed, conditions such as temperature and humidity will be more constant, providing more-appropriate conditions for virus survival. The amount of virus deposited will be more concentrated within a cattle shed than in outside areas of households, increasing the chance of detecting FMDV from the environment. Early work on FMD showed that the material on which FMDV is deposited can also affect virus survival. Experiments showed that when FMDV was dried on different materials, recovery of virus from hay and bran, in particular, favored survival over recovery from materials such as glass and cotton wool (12). In our study, wood, brick, and metal solid surfaces inside cattle sheds were more likely to produce FMDV-positive samples than surfaces in an outside environment. To retain the simplicity of our methods, environmental swabs were collected from solid surfaces, such as wooden posts and brick walls, that were deemed likely to have come into contact with secretions and excretions from an infected animal. Sample types such as hay or loose bedding are not as convenient to process under field conditions, one of the factors that influenced the design of our methods. Identifying those areas from which recovery of viral RNA is most likely focuses sampling efforts and maximizes the chances of recovering contaminating viral RNA from an environment. The exception to the rule that indoor sampling produces more FMDV RNA-positive samples than outside sampling is swabs collected from feed troughs in farm courtyards. All sampling for this study was carried out during daylight hours, when livestock are kept outside. Swabs collected from feed troughs in farm courtyards are therefore likely to capture virus shed more recently onto these surfaces than onto other surfaces. The use of electrostatic dust cloths as swabs enables reliable detection of FMDV RNA from solid surfaces that have been exposed to excretions and secretions from FMDV-infected animals. Collection of fecal samples for the detection of FMDV RNA proved unsuccessful, as suggested by previous work (13), although persistence of the virus in feces at low temperatures is possible, enabling survival in the environment.

Aerosolized FMDV has been detected in the breath of experimentally infected animals by use of a range of aerosol-sampling devices (14, 15). In cattle, the duration of viable virus emission in breath is around 4 to 5 days after the appearance of vesicular lesions (16). While the number of samples collected in this study is limited, the collection of FMDV-positive aerosol samples supports the use of air sampling as a possible surveillance method (17), especially for relatively large herds. Aerosol sampling enables investigators to sample animals gathered in an enclosed space by a noninvasive surveillance method on a herd level, rather than employing individual clinical examination (18, 19). The sensitivity of FMDV detection in air samples would need to be assessed when large numbers of animals were present. In this study, as expected, aerosol samples from cases older than 10 days were negative for FMDV, suggesting that, as with oral swabs, detection of FMDV by this method is most effective during the early stages of clinical disease (20, 21).

The production of VP1 sequence data, albeit from a single environmental sample, suggests that environmental sampling methods could be used in the surveillance of circulating strains rather than simply for determining whether FMDV is present. The difficulty in this will be generating sequence data from samples where genomic material is potentially degraded, to various degrees, depending on the environmental conditions to which virus particles have been exposed. Further characterization of samples with *C_T_* values below 30 will determine whether environmental samples are appropriate for the generation of sequence data. Limitations do exist in using environmental virus data to monitor circulating strains, since it would be difficult to determine how long virus particles had been present in the environment. This would be a particular issue where there are no clinical case histories to trace the source of environmental contamination. Further study concerning the survival limits of the virus and the viral genome in the environment would put more accurate limits on these data.

This study has demonstrated that FMDV RNA can be detected in the environment on infected premises in a setting of endemicity. Aerosol sampling of exhaled air from animals displaying clinical signs of the disease provides a further noninvasive method for the detection of FMDV. A key finding of this study is the demonstration that environmental sampling allows the detection of FMDV from environmental samples collected from premises where active clinical FMDV infection is not present. This extends the period during which FMDV can be confirmed as being present at a particular location. However, laboratory testing of samples is still a requirement to confirm the presence of the virus. For the development of the environmental sampling protocol, the use of pen-side or portable diagnostic tests (22, 23) would enable the rapid detection of the virus from environmental samples. In the case of an outbreak in a FMD-free country, where rapid detection is essential for appropriate control measures to be implemented, the conjunction of environmental sampling with pen-side diagnostics could create a useful tool for viral detection. A noninvasive sampling protocol, such as that described in this paper, could remove some of the burden of examination of large numbers of livestock by experienced veterinarians (24) and speed up the detection of suspected cases.

## MATERIALS AND METHODS

Locations in the Kathmandu Valley with active outbreaks of FMD were identified and reported by local veterinary technicians. Sites with these cases were then visited to confirm the presence of the disease by clinical inspection of livestock by experienced veterinarians. Sampling additional to that described here was used to confirm the diagnosis of FMD. The history of livestock movements and information regarding treatment or vaccinations was collected from livestock owners. The stage of disease was approximated by determining the ages of visible lesions (25). In November and December 2016, a total of 11 households over three sites were visited and sampled (Table 1), with return visits made to households 1 and 5 at 5 and 2 days after the initial sampling visit, respectively. Samples were collected from eight households at three sites in April and May 2017, and a return visit to household 14 was made 3 days after the initial visit. The third field visit, in November 2017, involved sample collections at three sites, with a total of five households. Household 21, at site 7, was revisited 1 week after the initial visit for the collection of further samples.

Electrostatic dust cloths (Minky, UK) were used to swab surfaces on premises deemed likely to have come into contact with excretions and secretions from infected animals. Feed buckets and troughs, wooden posts used to tether cattle, and surfaces inside animal shelters where virus secretions may have accumulated are examples of areas targeted for sampling. After the swabbing of an area, cloths were added to 5 ml of impinger fluid (Glasgow minimum essential medium [Gibco, UK] with antibiotics [penicillin-streptomycin and amphotericin B {Gibco, UK}], 5% bovine serum albumin [BSA; Sigma-Aldrich, UK], and 1 M HEPES [Gibco, UK]) in a screw-top pot, and the contents were shaken to fully saturate the cloth with the medium. A disposable wooden spatula was used to remove the cloth, and at the same time, it was pressed to extract as much medium as possible. An aliquot of medium was then added directly to a guanidinium thiocyanate-based lysis buffer (catalog no. AM8500; Thermo Fisher Scientific, UK) at a ratio of 1:2.6.

Fresh fecal samples were collected from the ground close to infected cattle where it was possible to visually link specimens to individual animals. A fecal suspension was made by adding the solid sample to 5 ml of impinger fluid. Large solid matter was removed by filtering the fecal suspension through muslin squares. Aliquots were collected from the resulting filtrate and were added directly to lysis buffer as described above.

Aerosol samples were collected using the Coriolis μ air sampler (Bertin Technologies), which functions as an impinger device. Airborne particulate matter is collected into 10 ml of impinger fluid (as described above) held in a collection vessel attached to the device. The Coriolis μ sampler was set at 300 liters/min and was run for 10 min for each aerosol sample collection. After each sampler run, aliquots of impinger fluid were added directly to the lysis buffer as described above.

Where possible, oral swabs were collected from infected animals to act as positive controls for virus detection. Cotton-tipped swabs were used to collect a sample from the front of the mouth and tongue of cattle. After collection, 2 ml impinger medium was added to the tube containing the oral swab. Swabs were left to elute in the medium for as long as possible at the collection site (approximately 20 min). Aliquots of medium from the oral swab were then added directly to the lysis buffer as described above.

All samples were stored at 4°C for as long as 33 weeks prior to transport to The Pirbright Institute for testing by real-time reverse transcription-PCR (rRT-PCR). Storage at either −20°C or −80°C might have improved detection rates from samples, but this was not available at the time. Viral RNA was extracted from samples using the KingFisher Flex automated extraction platform (Thermo Fisher Scientific, UK) with the MagMAX viral RNA isolation kit (Thermo Fisher Scientific, UK). FMDV RNA was detected by rRT-PCR on the ABI 7500 system (Applied Biosystems, UK) using a PCR that targets the polymerase 3D region of the FMDV genome (26).

The relationship between the maximum lesion age and the proportion of positive samples was assessed using a generalized linear model with quasi-binomial errors (to allow for overdispersion) and a logit link function. The response variable was the proportion of positive samples, and the explanatory variable was the lesion age, categorized as none, fresh (1 to 5 days), healing (6 to 10 days), or old (>10 days). The effects of sample type and location on the probability of an environmental sample being positive were explored using a generalized linear mixed model with binomial errors and a logit link function. The response variable was the sample status (positive or negative), and the explanatory variables were the sample type (brick, feed trough, ground, metal, or wood) and location (inside or outside), with an interaction between these as fixed effects and the household as a random effect.

The VP1 coding sequences from environmental samples were determined by following the Sanger dideoxy-sequencing methods as described previously (27).
